# Achilles tendon ultrasound-derived properties of the dominant and non-dominant jumping leg of university basketball athletes: relation with performance, range of motion, and injury

**DOI:** 10.3389/fspor.2026.1753505

**Published:** 2026-02-18

**Authors:** Owen Soontjens, Jordan Busner, Maryse Fortin

**Affiliations:** 1 Department of Health, Kinesiology and Applied Physiology, Concordia University, Montreal, QC, Canada; 2 School of Health, Concordia University, Montreal, QC, Canada

**Keywords:** achilles tendon, basketball, injury, performance, shear wave elasotography, ultrasound

## Abstract

**Introduction:**

The Achilles tendon (AT) plays a crucial role in force transmission and movement efficiency, and greater tendon stiffness may enhance elastic energy storage and improve performance in explosive movements. Shear-wave elastography (SWE) enables reliable, non-invasive assessment of tendon stiffness, yet its associations with other ultrasound (US)-derived properties and functional outcomes remain insufficiently defined in athletes. Limited normative data exists for university-level basketball players, despite the relevance of tendon adaptations and asymmetries in this population. Therefore, this study aimed to examine AT stiffness, thickness, echo intensity (EI), and fibrillar pattern in male and female university basketball athletes, and to explore their associations with ROM, injury history, and performance measures.

**Methods:**

Thirty-one university basketball athletes participated in this cross-sectional study. US imaging and SWE measured AT stiffness, thickness, and EI in both the dominant jumping leg (DJL) and the non-dominant jumping leg (NDJL). Functional assessments included a single-leg vertical jump, heel raise (HR) test, and ankle dorsiflexion ROM. Participants also provided demographic information and injury history data.

**Results:**

Female players had lower AT thickness compared to males and showed a trend toward lower stiffness. No significant differences in stiffness, thickness, or EI were observed between the dominant and non-dominant jumping legs. Male players with a prior lower-body injury exhibited lower AT stiffness. Correlation analyses revealed no consistent associations between AT properties and performance or ROM, although a strong negative correlation between AT thickness and EI emerged in both sexes.

**Discussion:**

Considering sex and injury history when interpreting tendon properties is crucial, and future larger-scale, longitudinal studies are needed to better understand how tendon characteristics evolve and inform training and injury prevention strategies.

## 1 Introduction

Tendons play a fundamental role in the musculoskeletal system. They are strong, flexible connective tissues that transfer muscular forces to bones to enable joint motion and contribute to movement efficiency (1–3). The Achilles tendon (AT), the largest and strongest tendon in the human body, connects the gastrocnemius and soleus muscles to the calcaneus and is essential for activities such as walking, running, and jumping (1, 4–6). Although the AT is a robust structure, it is highly susceptible to injury, particularly due to overuse in sports and physical activity, making it an essential focus of research among high-performance athletes (1, 7). Despite its well-established functional role, the methods used to assess its properties and how these relate to overall tendon health and performance are insufficiently defined.

Athletic populations exhibit greater AT stiffness than non-athletes, with variations observed across sport disciplines and competitive levels, likely reflecting physiological adaptations to repeated high-intensity loading (6, 8–13). Elevated stiffness is argued to enhance athletic performance by optimizing elastic energy storage and release during rapid movements such as sprinting, jumping, and changing direction (3, 8, 14–16). It may also improve the efficiency of the stretch-shortening cycle and contribute to a higher rate of force development (3, 8, 14, 15).

While greater functional stiffness has been shown to benefit performance, both excessive and insufficient stiffness may increase injury risk. Excessive local stiffness can increase peak forces and limit joint mobility, whereas reduced stiffness may allow excessive motion, potentially leading to soft tissue injuries (3, 8, 14, 15, 17). In line with this interplay, studies investigating the relationship between AT stiffness and ankle range of motion (ROM) have reported an inverse association with greater stiffness linked to less ROM, which is also argued to be a risk factor for injury (18–20).

Beyond absolute stiffness, asymmetry between limbs may also be clinically relevant. Findings on AT stiffness asymmetry index (AI) between the dominant jumping leg (DJL) and non-dominant jumping leg (NDJL) remain inconclusive (21, 22). However, given that jumping performance asymmetries exceeding 10% have been linked to increased injury risk (21), further investigation into AT stiffness asymmetries and their relationship to jumping performance is warranted. This is particularly relevant in basketball athletes, where single-leg jumping is fundamental and notable differences in performance between the dominant and non-dominant legs have been reported (21).

Since tendon adaptations are both mechanical and structural, other ultrasound (US)-derived characteristics such as thickness, echo intensity (EI), and fibrillar organization provide complementary insights. Alterations in one parameter are frequently accompanied by changes in the others, particularly in injured or pathological conditions (23–25). Injured tendons typically exhibit reduced stiffness, increased thickness, and disrupted fibrillar architecture, with lower EI reflecting this structural disorganization (23–25). These properties may also interact with one another, as an increase in tendon thickness may reflect a compensatory response to localized structural disruption (24). However, the extent to which these imaging-derived variables are associated with functional outcomes such as performance, ROM, and injury risk remains unclear in athletes.

Advancements in imaging technologies have introduced shear wave elastography (SWE) as a non-invasive and objective method to quantify tissue stiffness by measuring the speed of shear waves (26–32). By assessing shear wave propagation speed as a proxy for tissue stiffness, SWE enables reproducible evaluation of tendon mechanical properties (26, 28, 29, 33, 34). Research utilizing SWE as a tool to analyze the AT has steadily grown, with studies demonstrating the technique's reliability in both healthy and pathological participants (28, 29, 35, 36).

While SWE holds promise for assessing tendon properties, accurate and reliable measurements require strict control of methodological variables such as joint position, recent physical activity, region of interest (ROI) size, probe frequency, and movement artifacts (22, 27, 37–42). Variability in these factors can significantly affect SWE readings and subsequent interpretation. Despite its potential, limited data exist on normative AT stiffness values in university-level athletes and on how stiffness relates to other US-derived variables, performance metrics, ROM, and injury Hx (3, 6, 8, 14, 15). Establishing baseline values and clarifying these relationships is essential for optimizing training, monitoring, and rehabilitation strategies.

Therefore, the current study aimed to investigate AT stiffness, thickness, EI, and fibrillar pattern among male and female university-level basketball players. Specifically, we examined 1) the differences between the DJL and the NDJL, and 2) possible associations between these tendon characteristics and ankle ROM, injury Hx, single-leg jump performance, and heel raise (HR) test performance.

## 2 Materials and methods

### 2.1 Study design

This cross-sectional study was reported in accordance with the STROBE guidelines (43).

### 2.2 Participants

Eligibility criteria were defined *a priori*. To be included in the study, participants were required to meet all of the following criteria:

- Be listed on the Concordia University varsity basketball team roster for the 2024–2025 competitive season
- Be 18 years of age or older
- Provide written informed consent to undergo US and SWE imaging of the AT on both limbs and complete all subsequent functional and practical testing procedures

Participants were excluded if they met any of the following criteria:

- History of surgical intervention involving the AT
- Recent lower-limb surgery or injury that precluded safe participation in the practical testing procedures

A total of 33 basketball players (21 males, 12 females) from Concordia University varsity teams volunteered to participate in this study. One female player was excluded due to prior reconstructive surgery to the AT, and one male player was excluded due to a recent anterior cruciate ligament surgery, which incapacitated them from undergoing the practical test. This resulted in a final sample of 31 players (20 males, 11 females), representing 95% of the men's roster and 92% of the women's roster. The study was approved by the Central Ethics Research Committee of the Quebec Minister of Health and Social Services. All players provided informed consent prior to the assessment.

### 2.3 Measurements

All participants completed the tests in the same order: questionnaires, US imaging and SWE, ankle dorsiflexion ROM assessment, single-leg jump, and finally the HR test.

#### 2.3.1 Questionnaires

An initial survey was conducted to gather demographic details, including the participants' age, sex, ethnicity, height, weight, years of playing experience, and position. Evaluation of current AT pain and functionality was conducted using the Victorian Institute of Sport Assessment-Achilles (VISA-A). This questionnaire serves as a reliable assessment tool for the AT (44). Participants then completed a detailed questionnaire assessing their lower limb injury history (Hx). The questionnaire instructed them to report all lower-body injuries they had sustained that had prevented them from participating in their sport in the 12 months preceding the completion of the questionnaire. The players were also required to indicate the injury location, duration, and whether the injury was recurrent or isolated.

#### 2.3.2 Ultrasound imaging and shear wave elastography

Participants underwent US evaluation to assess thickness, EI, fibrillar pattern, and resting stiffness of the AT using Concordia University School of Health's Aixplorer US unit (Supersonic Image, Aix-en-Provence, France; software version V12.2.0.808) equipped with a high-frequency linear array transducer (SL 15–4, 4–15 MHz). Tendon stiffness was primarily quantified as Young's modulus (kPa) as provided by the Aixplorer SWE software. Shear wave speed (SWS) (m/s) was additionally recorded for descriptive purposes, but all subsequent analyses were performed using Young's modulus. US imaging and SWE assessments were performed in accordance with international musculoskeletal US guidelines published by the European Federation of Societies for Ultrasound in Medicine and Biology (EFSUMB) (45, 46). US examinations and measurements were performed by a single trained sonographer (master's-level researcher) who completed multiple supervised training sessions with experienced musculoskeletal sonographers and conducted test–retest assessments on randomly selected participants to ensure measurement reliability. The system was operated using a standardized Musculoskeletal—Foot and Ankle preset, with imaging depth set to 2.0 cm and the SWE full-scale range set to 600 kPa to avoid signal saturation. Overall gain and image-processing parameters were optimized at the beginning of data collection and maintained constant across all participants, and the focal zone was consistently positioned at the level of the AT. All measurements were taken between October and December 2024 in the same room at Concordia University's School of Health, with room temperature regulated at 21 degrees Celsius.

**Figure 1:**
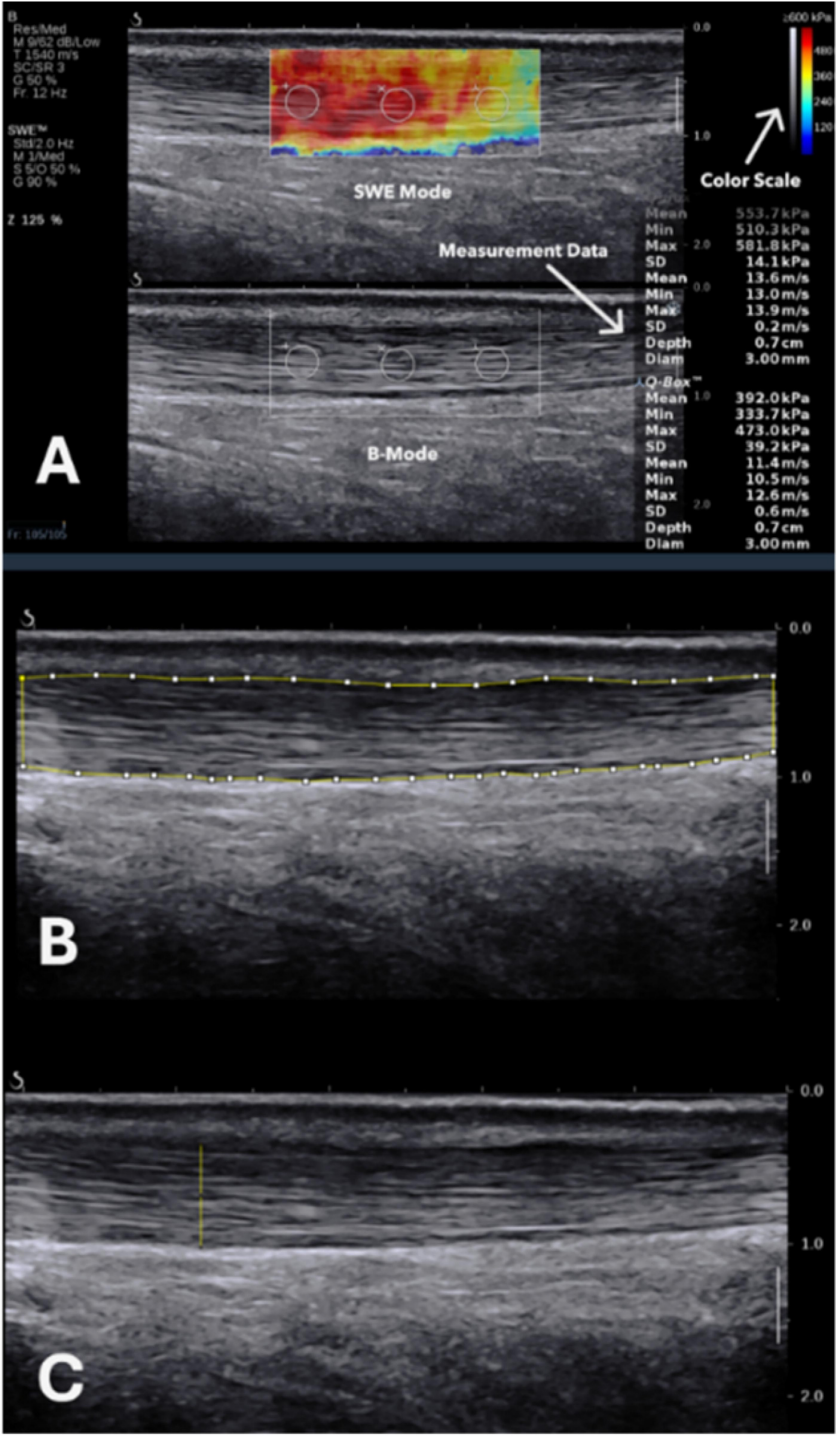
**(A)** B-mode identification of the AT and SWE measurement. **(B)** Tracing of the AT for EI measurement. **(C)** AT maximal thickness measurement.

The players were instructed not to engage in any form of training on the day of assessment before the measurements to ensure reliable and precise AT stiffness measurements (35, 47). Shoes and socks were removed to allow for the probe's direct access to the skin. Players were then instructed to lie prone with their feet hanging off the edge of the examination table in a relaxed position to ensure more reliable results (29). Gel was applied to the targeted area, and the high-frequency linear probe was aligned along the axis of the AT. Consistent light pressure was maintained during the measurements, with both the evaluator and the participant minimizing their movements. AT was identified and pictured in B-mode by placing the transducer on the posterior aspect of the lower limb, 3 cm proximal to the tendon's insertion onto the calcaneus. At this location, three images were captured on each side for the analysis of tendon thickness, EI, and fibrillar pattern. This site was chosen due to its anatomical relevance, avoidance of saturation, and reproducibility reasons. The tendon's mid portion (2–6 cm from the calcaneus) is considered the avascular zone of the tendon. It is commonly affected in tendinopathy and is most relevant to clinical pathology, as it presents significant variations in SWE readings when affected (28). The distal tendon shows very high SWE measurements, and in athletes who already have elevated AT stiffness, measurements taken closer to the calcaneus may approach or even exceed the Aixplorer's maximum detectable limit (16.3 m/s) (4). Saturation and reduced measurement accuracy are avoided by taking measurements at the midportion. After the target region was identified and imaged, the evaluator switched to SWE mode, allowed a brief stabilization period (approximately 3–5 s) to ensure signal stability, froze the image, and proceeded to obtain SWE measurements using a constant round ROI of 3 mm in diameter (Figure 1). The constant 3 mm circular ROIs are in accordance with previous research (36, 40), and were used to standardize sampling. These were positioned within the mid-portion of the tendon core to avoid tendon borders and visible artefacts or heterogeneous regions on the elastography map. Three measurements were obtained per side and averaged to reduce pixel-level variability. The mean was calculated and used in the analysis.

The analysis of AT thickness, EI, and fibrillar pattern was conducted retrospectively using the captured images. Thickness and EI were quantified with ImageJ software (National Institutes of Health, Bethesda, MD, USA). Thickness was determined as the mean of the thickest segment measured across the three images, in accordance with previously established methodologies (48, 49). For each longitudinal B-mode image, tendon thickness was measured in *ImageJ* as the maximum anteroposterior distance between the superficial and deep tendon borders, using a perpendicular caliper placed at the visually identified thickest point along the imaged tendon segment. EI was calculated as the mean grayscale value of the manually traced tendon across the three images; this value for a pixel could range from 0 (dark) to 255 (bright), adapted from previously employed methodologies (50). Fibrillar pattern was evaluated visually for each tendon by the investigator and categorized as one of either fine, parallel, and linear fibrillar pattern or disrupted, fragmented, and absent fibrillar pattern, adapted from established categorization techniques (24, 51).

#### 2.3.3 Performance

##### 2.3.3.1 Running single leg vertical jump test

The running single-leg vertical jump test using a Vertec device is a field-based assessment of unilateral lower-limb jumping performance, requiring participants to jump off one leg following a standardized running approach and reach vertically at peak jump height. The Vertec tool is recognized for its sensitivity in measuring functional performance asymmetry between lower limbs and is commonly used in physical education and sports settings due to its practicality and cost-effectiveness (52).

Participants were required to wear standard athletic clothing and their basketball shoes. To ensure consistency, all tests were conducted in the same environment and supervised by the same evaluator.

Before the jump protocol, players' standing reach height was measured. The players were instructed to stand sideways next to a wall with their feet together, their heads in a neutral position, and their eyes looking straight ahead. They then raised the hand closest to the wall as high as possible, keeping it against the wall, and took a deep breath. The measurement was taken at the peak of the deep breath, from floor level to the highest point reached, and recorded to the nearest 0.1 cm. This was repeated on the opposite side to account for potential differences in limb length and shoulder ROM.

A standardized warm-up session, lasting 5–10 min, was then conducted to optimize participant performance and minimize injury risk (53, 54). This included a treadmill run, deep squats, double-leg jumps, and lower-body dynamic stretching.

**Figure 2:**
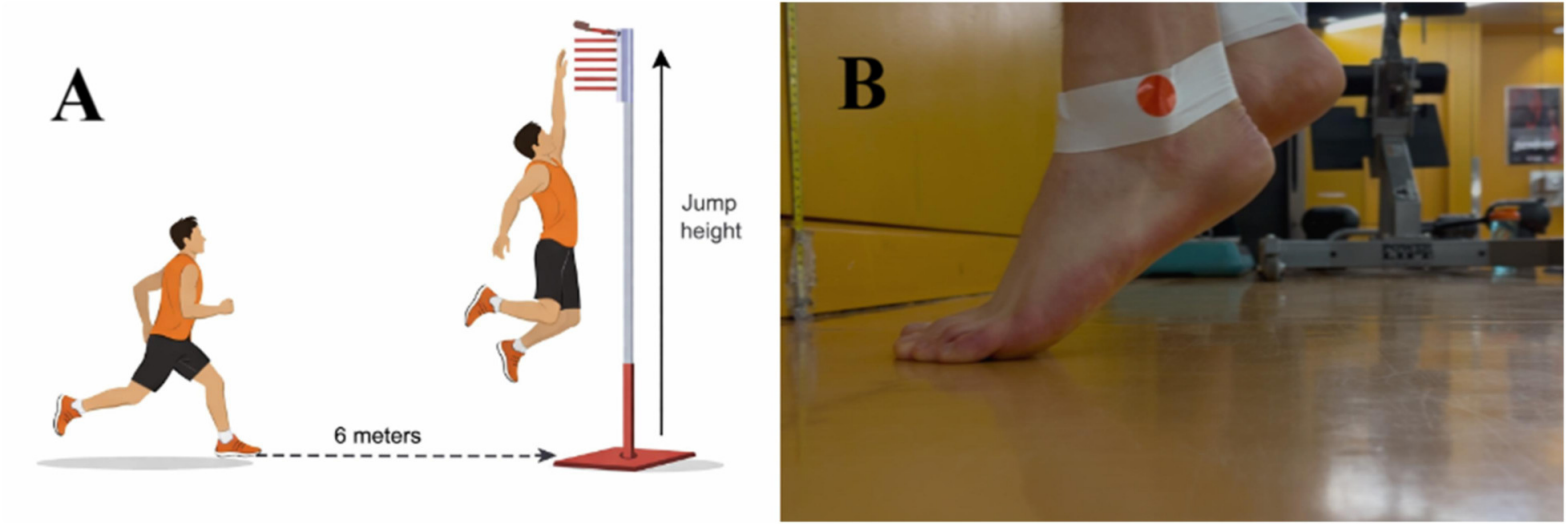
**A:** running single-leg vertical jump test performed using a vertec device. **B:** HR test performed with athletic tape and a red sticker as a tracking marker.

Following the warm-up session, participants received verbal instructions and a demonstration of the test procedure. They were allowed to practice once with the Vertec tool to gain confidence in performing the task. To replicate basketball conditions, participants approached the Vertec from 6 meters away, equivalent to the distance of the 3-point line on a basketball court, and performed a maximal running single-leg jump to reach the highest Vertec swivel vane attainable at full arm extension (Figure 2A). Each participant performed three jumps per side, alternating jumping leg between each trial to minimize potential fatigue effects on subsequent jump performance. The highest jump attempt from the three trials was recorded for each leg. The recorded score was calculated as the difference between standing reach height and the maximum jump height achieved for each leg (21).

##### 2.3.3.2 Heel raise test

While the running single-leg vertical jump test closely simulates basketball-specific conditions, it requires precise coordination of both lower and upper body movements to achieve optimal scores. To perform the jump test correctly, athletes must extend their arms upward, aiming to touch the highest swivel vane precisely at the peak of their leap, which is a significantly complex task.

The heel-raise test is a method used for evaluating plantar flexion strength and specifically targets the AT (55). This test was selected to provide a complementary, tendon-specific functional assessment involving repeated cyclic loading of the AT, allowing exploration of potential associations between AT mechanical properties and sustained plantar flexor performance. Much like the Vertec tool, this test is reliable and cost-effective for assessing performance (55, 56). Recent refinements to the HR test protocol emphasize measuring heel-raise height in addition to repetition count, providing a more comprehensive estimate of functional capacity (55, 56).

Participants stood approximately 15 cm away from a wall, facing it, with two fingers of each hand placed on the wall at shoulder height and width apart for balance. The evaluator ensured that only minimal pressure was applied to the wall. Once properly positioned, a strip of white athletic tape was wrapped around both ankles, and a red circular sticker (1.5 cm in diameter) was fixed to the most prominent part of each lateral malleolus. A camera was placed at floor level beside the participant, who was then instructed to lift the foot farthest from the camera off the ground (Figure 2B).

Upon starting the recording, participants performed single-leg HR in sync with a metronome set to 1 beat per second, corresponding to two beats per full heel-raise cycle (ascent and descent) (55). They were instructed to complete as many repetitions as possible, aiming for maximum height. A successful repetition required maintaining the set pace and minimizing fingertip contact with the wall for balance. Participants received a warning if they failed to meet these criteria, and the test concluded when they were unable to complete two consecutive successful repetitions (57). The assessment was conducted bilaterally.

Video analysis was conducted using the previously validated “Calf Raise” mobile application (58). The recorded videos were uploaded to the app, which measured key performance metrics, including repetition count, maximum height, total height, fatigue percentage, positive and negative work, and positive and negative power. To enable analysis, a tracking circle was adjusted around the red sticker, allowing the app to process the data accurately.

#### 2.3.4 Dominant vs. non-dominant jumping leg

Participants' dominant jumping side was determined by their single-leg vertical jump test results, with the better-performing side identified as the DJL.

#### 2.3.5 Ankle range of motion

The distance-to-wall technique was used in a weight-bearing lunge position to measure ankle joint dorsiflexion. Previous studies have found that this technique is more accurate than non-weight-bearing methods in assessing ROM (59). It is cost-effective and eliminates the need for specialized equipment, which makes it suitable for reliable execution by novice evaluators (59). Participants were instructed to touch the wall with two fingers from each hand to maintain stability while performing a forward lunge. With the test foot placed perpendicular to the wall, participants lunged forward until their front knee made gentle contact with the wall while ensuring the front foot remained flat on the ground. Subsequently, the testing foot was gradually shifted away from the wall until the knee barely touched it. Ankle dorsiflexion ROM measurement was identified as the maximum distance between the big toe and the wall while maintaining knee-wall contact and without lifting the heel.

### 2.4 Statistical analysis

Means, medians, and standard deviations were calculated for athlete demographics, AT stiffness, thickness, and EI, running single-leg vertical jump test, heel-raise test, ankle ROM, and injury Hx. To determine the AI percentage in stiffness and jump height, this formula was used:

$$\left(\frac{\mathrm{Larger}-\mathrm{Smaller}}{\mathrm{Larger}}\right)\times 100$$

Normality of the data was assessed and confirmed using the Shapiro–Wilk test and Kolmogorov–Smirnov test as well as visual inspection of histograms and Q–Q plots. Paired and independent t-tests compared AT stiffness, thickness and EI between the DJL and NDJL, injured and non-injured, male and female athletes, as well as between disrupted and normal fibrillar pattern tendon types. Pearson correlation analyses were conducted to determine possible correlations between the US imaging-derived measures and performance, as well as ankle ROM. The correlation strength was interpreted accordingly to Cohen's guidelines (60): 0.1–0.2 weak correlation, 0.3–0.5 moderate correlation, and >0.5 strong correlation. All statistical analyses were performed separately for male and female athletes. Statistical significance was defined as *p* < 0.05, and all analyses were conducted using IBM SPSS Statistics for Windows (Version 30.0.0.0; IBM Corp., Armonk, NY, USA).

## 3 Results

All variables met assumptions of normality based on Shapiro–Wilk and Kolmogorov–Smirnov tests (*p* > 0.05) and visual inspection of histograms and Q–Q plots. Parametric analyses were therefore applied.

The characteristics of the players who participated in the study are summarized in Table 1. The average (mean ± SD) age, height, and weight of the participants were 22.19 ± 1.87 years, 185.09 ± 12.02 cm, and 82.44 ± 12.31 kg, respectively. On average, participants had 9 years of experience playing competitive basketball, including 2 years at the university level. Additionally, 65% (*n* = 20) of players reported experiencing at least one lower-limb injury within the past 12 months. Table 2 presents the participants' results in the functional tests.

**Table 1:** Participants' characteristics.

Variable	All (*n* = 31)	Female (*n* = 11)	Male (*n* = 20)
Age (yr)	22.2 ± 1.9	22.5 ± 2.1	22.0 ± 1.8
Height (cm)	185.1 ± 12.0	173.0 ± 8.2	191.8 ± 7.8
Weight (kg)	82.4 ± 12.3	71.2 ± 8.6	88.6 ± 9.3
Dominant jumping leg (*n*)			
Right	13	6	7
Left	18	5	13
Playing position (*n*)			
Point guard	6	3	3
Shooting guard	10	4	6
Small forward	6	1	5
Power forward	4	2	2
Center	5	1	4
Basketball competitive level (yr)	9.0 ± 2.8	8.9 ± 3.3	9.1 ± 2.5
Basketball university level (yr)	2.3 ± 1.3	2.2 ± 1.3	2.4 ± 1.3
Participants reporting injuries (*n*)	20	6	14
Lower-body injuries reported (*n*)	24	6	18
Recurrent injuries	12	2	10
Isolated injuries	12	4	8
Lower-body injury location (*n*)			
Hip	2	0	2
Ankle/foot	14	4	10
Knee	6	2	4
Calf/shin	2	0	2
VISA-A score (/100)	98.2 ± 5.2	95.6 ± 8.3	99.7 ± 0.7

**Table 2:** ROM and performance test results.

Variable	All (*n* = 31)	Female (*n* = 11)	Male (*n* = 20)
DJL ankle dorsiflexion ROM (cm)	10.1 ± 3.7	10.2 ± 2.2	10.0 ± 4.3
NDJL ankle dorsiflexion ROM (cm)	10.0 ± 7.0	10.1 ± 3.5	9.9 ± 4.3
DJL jump height (cm)	67.8 ± 15.5	48.0 ± 5.4	76.6 ± 8.8
NDJL jump height (cm)	61.3 ± 13.9	44.3 ± 4.8	69.0 ± 8.6
DJL heel-raise repetitions (n)	31 ± 10	35 ± 15	29 ± 7
NDJL heel-raise repetitions (n)	30 ± 7	31 ± 10	29 ± 5
DJL heel-raise height (cm)	199.0 ± 75.4	179.5 ± 101.6	207.8 ± 61.4
NDJL heel-raise height (cm)	198.0 ± 60.8	168.3 ± 66.5	211.3 ± 54.6

### 3.1 Male & female AT characteristics

Table 3 presents AT stiffness expressed as Young's modulus (kPa), SWS (m/s), thickness, and EI measurements for the males and females in the DJL and NDJL, along with the AT stiffness AI. Female players exhibited lower thickness (*p* = 0.011) values compared to their male counterparts. No significant differences were found in AT stiffness AI between legs, in either male or female players (*p* = 0.592).

**Table 3:** Mean tendon structural values in male and female players.

Variable	Male (*n* = 20)	Female (*n* = 11)	Mean difference (95% CI)	*p*-value
Stiffness (Young's modulus, kPa)	455.4 ± 72.4	411.5 ± 50.9	43.9 (−6.6, 94.4)	.086
Stiffness (SWS, m/s)	12.2 ± 1.0	11.6 ± 0.7	0.6 (−0.1, 1.3)	.084
DJL stiffness (kPa)	448.2 ± 83.6	406.3 ± 41.5	41.9 (−4.1, 87.9)	.073
NDJL stiffness (kPa)	462.6 ± 84.6	416.6 ± 81.0	46.0 (−18.0, 110.0)	.153
Stiffness asymmetry (%)	13.3 ± 10.4	11.2 ± 9.2	2.0 (−5.7, 9.7)	.592
Mean thickness (cm)	0.58 ± 0.06	0.52 ± 0.06	0.06 (0.02, 0.11)	.011
DJL thickness (cm)	0.58 ± 0.06	0.53 ± 0.07	0.05 (−0.00, 0.10)	.052
NDJL thickness (cm)	0.58 ± 0.06	0.50 ± 0.06	0.07 (0.03, 0.12)	.004
Mean echointensity (A.U.)	76.3 ± 16.0	91.1 ± 25.9	−14.8 (−33.2, 3.6)	.108
DJL echointensity (A.U.)	79.2 ± 21.7	88.6 ± 21.8	−9.4 (−26.1, 7.3)	.260
NDJL echointensity (A.U.)	73.5 ± 18.1	93.6 ± 37.1	−20.1 (−45.9, 5.6)	.115

Stiffness was primarily quantified as Young's modulus (kPa) and used for all statistical analyses. SWS (m/s) is presented for descriptive purposes only.

### 3.2 DJL & NDJL AT characteristics

While the NDJL exhibited slightly greater stiffness than the DJL, the difference did not reach statistical significance in either males (*p* = 0.461) or females (*p* = 0.672), as shown in Table 4. AT thickness values in the DJL and NDJL in males were nearly identical (*p* = 0.795), while thickness in the DJL in females was marginally greater than in the contralateral side, though not statistically significant (*p* = 0.056, Table 4). No differences in EI were found between the two sides in either males or females (*p* = 0.300 and *p* = 0.614, respectively, Table 4).

**Table 4:** DJL and NDJL AT properties by sex.

Sex	Variable	DJL	NDJL	Mean difference (95% CI)	*p*-value
Male (*n* = 20)	Stiffness (kPa)	448.2 ± 83.6	462.6 ± 84.6	−14.4 (−54.4, 25.6)	.461
	Thickness (cm)	0.58 ± 0.06	0.58 ± 0.06	0.00 (−0.02, 0.02)	.795
	Echointensity (A.U.)	79.2 ± 21.7	73.5 ± 18.1	5.8 (−5.5, 17.0)	.300
Female (*n* = 11)	Stiffness (kPa)	406.3 ± 41.5	416.6 ± 81.0	−10.4 (−63.3, 42.6)	.672
	Thickness (cm)	0.53 ± 0.07	0.50 ± 0.06	0.03 (−0.00, 0.06)	.056
	Echointensity (A.U.)	88.6 ± 21.8	93.6 ± 37.1	−5.0 (−26.4, 16.4)	.614

### 3.3 AT stiffness characteristics in relation to history of injury

Stiffness, thickness, and EI measurements for players with and without a reported lower-body injury in the previous 12 months are presented in Table 5. On average, male players who reported an injury had a mean stiffness that was 71.12 kPa lower compared to their non-injured counterparts (*p* = 0.040). No differences were found in AT thickness or EI between the previously injured group and non-injured group in males and females (Table 5).

**Table 5:** AT properties according to history of lower-body injury, stratified by sex.

Sex	Variable	No hx of injury	Hx of injury	Mean difference (95% CI)	*p*-value
Male	Stiffness (kPa)	505.2 ± 79.9	434.1 ± 59.7	71.1 (3.5, 138.7)	.040
	Thickness (cm)	0.55 ± 0.05	0.58 ± 0.06	−0.03 (−0.09, 0.03)	.260
	Echointensity (A.U.)	81.3 ± 20.8	74.2 ± 13.8	7.2 (−9.3, 23.6)	.372
Female	Stiffness (kPa)	427.6 ± 67.1	398.0 ± 33.1	29.7 (−40.3, 99.6)	.363
	Thickness (cm)	0.54 ± 0.08	0.50 ± 0.04	0.05 (−0.03, 0.13)	.210
	Echointensity (A.U.)	84.4 ± 33.8	96.7 ± 18.6	−12.4 (−48.6, 23.9)	.460

### 3.4 AT US properties and functional measures in relation to fibrillar pattern

AT properties and functional measurements, stratified by fibrillar pattern integrity, are summarized in Tables 6, 7. Overall, 30% of the analyzed tendons were classified as exhibiting disrupted fibrillar patterns. Among male players, 13 ATs demonstrated fibrillar disruption, while 27 were categorized as normal. In female players, 6 ATs displayed disruption and 16 were normal. No significant differences were observed in structural or functional parameters between tendons with normal vs. disrupted fibrillar patterns, with several values being nearly identical across groups.

**Table 6:** Male fibrillar pattern characteristics' comparison.

Variable	Disrupted fibrillar pattern	Normal fibrillar pattern	Mean difference (95% CI)	*p*-value
Stiffness (kPa)	466.8 ± 64.1	450.0 ± 91.8	16.8 (−34.2, 67.8)	.507
Thickness (cm)	0.58 ± 0.05	0.58 ± 0.07	0.00 (−0.04, 0.04)	.965
Echointensity (A.U.)	77.6 ± 20.0	75.8 ± 20.3	1.8 (−12.0, 15.6)	.793
Ankle dorsiflexion ROM (cm)	11.6 ± 4.4	9.2 ± 4.0	2.4 (−0.4, 5.4)	.094
Jump height (cm)	74.3 ± 12.6	72.1 ± 7.6	2.2 (−4.3, 8.7)	.493
Heel-raise height (cm)	210.2 ± 37.4	209.3 ± 65.5	0.9 (−38.4, 40.6)	.966
Heel-raise repetitions (*n*)	30 ± 6	29 ± 6	1 (−3, 5)	.695

**Table 7:** Female fibrillar pattern characteristics' comparison.

Variable	Disrupted fibrillar pattern	Normal fibrillar pattern	Mean difference (95% CI)	*p*-value
Stiffness (kPa)	382.6 ± 36.8	422.3 ± 68.2	−39.6 (−101.4, 22.1)	.196
Thickness (cm)	0.52 ± 0.03	0.52 ± 0.08	0.00 (−0.05, 0.05)	.993
Echointensity (A.U.)	102.1 ± 27.8	87.0 ± 30.3	15.1 (−14.6, 44.8)	.301
Ankle dorsiflexion ROM (cm)	9.7 ± 3.1	10.3 ± 2.8	−0.6 (−3.5, 2.3)	.673
Jump height (cm)	44.1 ± 9.8	46.6 ± 4.5	−2.6 (−9.9, 4.7)	.468
Heel-raise height (cm)	214.1 ± 81.1	165.9 ± 84.4	48.2 (−64.4, 160.8)	.377
Heel-raise repetitions (*n*)	38 ± 15	32 ± 13	6 (−11, 24)	.464

### 3.5 Correlations between AT properties and functional measures in male players

Table 8 presents the correlations between AT properties: stiffness, thickness, and EI, and functional outcomes, including mean ankle dorsiflexion ROM, HR mean total repetitions and height, and mean jump height in males. The only statistically significant association of interest observed was a strong negative correlation between average AT thickness and EI (*p* = 0.002).

**Table 8:** Correlation matrix between male AT properties and functional measures.

	1	2	3	4	5	6	7	8	9	10	11
Age											
Height	−0.26										
Weight	−0.17	.80^b^									
Stiffness	0.11	−0.10	−0.01								
Stiffness AI	0.33	−0.05	−0.31	−0.26							
Thickness	−0.05	0.05	−0.18	0.01	0.24						
Echo Intensity	−0.01	0.04	0.00	0.29	−0.07	-.63^b^					
Visa-a Score	0.13	0.19	0.13	−0.36	0.28	0.03	0.00				
ROM	−0.22	−0.05	−0.03	0.16	−0.30	−0.36	0.23	0.04			
HR Reps	-.48^a^	0.09	0.15	−0.35	−0.33	−0.15	0.06	−0.26	−0.19		
HR Height	-.56^b^	0.33	0.37	−0.20	−0.13	0.15	−0.04	−0.20	−0.18	.78^b^	
Jump Height	−0.25	0.00	−0.01	0.10	0.14	−0.17	0.11	0.14	0.21	0.14	0.16

a Correlation is significant at the 0.05 level (2-tailed).

b Correlation is significant at the 0.01 level (2-tailed).

### 3.6 Correlations between AT properties and functional measures in female players

Table 9 presents the correlations between the female AT properties and functional outcomes. Similarly, they displayed a strong negative correlation between AT thickness and EI (*p* = 0.034). A strong negative correlation was also found between mean EI values and stiffness (*p* = 0.049), and a strong positive correlation between thickness and stiffness AI (*p* = 0.036).

**Table 9:** Correlation matrix between female AT properties and functional measures.

	1	2	3	4	5	6	7	8	9	10	11
Age											
Height	0.58										
Weight	0.23	0.54									
Stiffness	−0.04	0.10	0.31								
Stiffness AI	−0.35	0.37	0.50	0.57							
Thickness	−0.20	0.19	0.57	0.25	.63^a^						
Echo Intensity	−0.08	−0.23	-.66^a^	-.60^a^	−0.55	-.64^a^					
Visa-a Score	−0.29	−0.01	−0.50	0.23	0.29	0.07	0.02				
ROM	0.21	.69^a^	0.08	0.19	0.29	−0.18	0.04	0.46			
HR Reps	0.26	−0.16	−0.12	−0.32	−0.44	0.30	−0.06	0.23	−0.26		
HR Height	0.12	−0.17	−0.22	−0.33	−0.40	0.18	0.06	0.20	−0.19	.86^b^	
Jump Height	0.00	0.40	0.30	−0.24	0.36	−0.12	0.21	−0.60	0.20	−0.63	−0.53

a Correlation is significant at the 0.05 level (2-tailed).

b Correlation is significant at the 0.01 level (2-tailed).

## 4 Discussion

### 4.1 Main findings

This cross-sectional study aimed to compare AT US-derived properties between the DJL and NDJL in male and female basketball players at the university level, and to examine their associations with jump and HR performance, ankle ROM, and injury Hx. Overall, there were no significant differences in stiffness, thickness, or EI between limbs, and no significant correlations were found between AT stiffness, thickness, or EI, and ankle ROM, or performance outcomes.

Despite the absence of significant group-level effects, several noteworthy trends emerged. Male players had thicker ATs and tended to have greater AT stiffness than female players. Injury Hx also appeared to influence AT stiffness: male players with a previous injury demonstrated lower mean stiffness than their non-injured counterparts, while previously injured female players exhibited lower AI than uninjured females. Among previously injured players, males with isolated injuries had greater stiffness AI than those with recurrent injuries. Finally, for both male and female players, a strong negative correlation was found between EI and thickness.

The stiffness, thickness and EI values observed in our study are consistent with those reported in other athletic populations. For young, healthy male athletes, a recent study reported a mean AT stiffness value of 12.19 m/s, which aligns with our findings of 12.23 ± 0.99 m/s in our male players (11). Similarly, the same research reported a mean value of 10.98 m/s for young, healthy female athletes, comparable to our results of 11.62 ± 0.69 m/s in our female players (11). With respect to morphology, maximal sagittal-plane tendon thickness was previously reported as 5.7 ± 1.0 mm in a cohort of 31 asymptomatic recreational cross-country runners (48), while mean EI at the midportion of the AT was found to be 89.85 ± 8.93 in 18 young, healthy volunteers (50). Both parameters are consistent with the values obtained in our study.

#### 4.1.1 Differences in AT stiffness, thickness, and EI between male and female players

Our findings indicate that males exhibit greater AT thickness and a tendency toward increased stiffness compared with females, although the latter did not reach statistical significance. These observations are consistent with prior reports in the literature (11, 49, 61–63). Such differences likely reflect inherent physiological variations between sexes, particularly in muscle mass and hormonal profiles, which influence AT properties (2, 62, 64). Tendons adapt to mechanical loading by increasing stiffness and thickness to accommodate the forces transmitted through the muscle-tendon unit (64). Since males generally exhibit greater body mass and force production (65), the resulting mechanical stress likely drives these adaptive changes.

Reduced AT cross-sectional compliance, which is a measure of the tendon's deformation across its thickness when loaded, may also explain the increased stiffness observed in males (61). Male tendons exhibit less elongation under equivalent loading, which indicates a diminished capacity for deformation and thus greater resistance to stretch (61, 62). In contrast, females typically demonstrate higher AT compliance, which may enhance adaptability to mechanical loading and is associated with lower tendon stiffness (61). Females also display elevated estrogen levels, which have been linked to altered collagen synthesis and show a negative association with tendon stiffness (62, 66).

While structural and mechanical sex differences in the AT are increasingly well-documented, imaging characteristics such as EI remain less explored. To date, no study has specifically examined sex differences in AT EI among athletes. Previous research, however, has reported lower EI values in the gastrocnemius and quadriceps of males compared with females (67). These differences are likely attributable to the higher proportion of lean muscle mass in males, characterized by a greater content of contractile tissue and water, which manifests as darker ultrasonographic images (67).

#### 4.1.2 Differences in AT stiffness, thickness, and EI between the DJL and NDJL

Consistent with previous research, our findings suggest that AT stiffness is not influenced by leg dominance (2, 22). While no studies to date have examined sagittal-plane differences in AT thickness between the DJL and NDJL in basketball players, prior investigations provide relevant insights. Specifically, no significant differences in AT thickness were observed between the right and left limbs of semi-professional running athletes (6), nor between the dominant and non-dominant sides in healthy young adults when assessed in the transverse plane (68). Similarly, to our knowledge, no studies have evaluated EI asymmetries between dominant and non-dominant ATs in performance-based athletes.

Although a previous study on university-level basketball players reported a significant increase in jump height of the dominant side over the other (21), our results indicate that the performance asymmetry is not associated with differences in AT stiffness, thickness or EI. Instead, these discrepancies in performance may be explained by ankle joint kinematics. Specifically, the dominant leg has demonstrated shorter ground contact times, larger ankle joint angles, and greater angular velocities than the non-dominant leg (21). While jump performance between the two legs was not associated with any of the US derived properties, 35% (*n* = 11) of the players demonstrated a jump height asymmetry greater than 10%, which has been argued to increase the risk of injury (21). This suggests that neuromuscular or biomechanical factors beyond tendon structure likely underlie functional asymmetries.

It is also important to note that our study employed a different definition of leg dominance than certain previous studies (2, 18, 22). The DJL was defined as the leg that produced the highest running single-leg jump height. This objective, performance-based criterion was selected because basketball players frequently display unilateral jump asymmetries and often struggle to identify their dominant leg subjectively. By reducing subjectivity, this approach provided a more standardized method for distinguishing the DJL from the NDJL.

#### 4.1.3 Relation between history of injury and AT stiffness, thickness, and EI

Our findings suggest that a recent Hx of injury may be associated with differences in AT stiffness in university-level athletes. Reported injuries affected the ankle, knee, hip, and shins, with ankle and foot injuries being the most prevalent (58% of all cases). Previously injured male players exhibited reduced stiffness compared to their non-injured counterparts. However, these findings should be interpreted with caution due to the relatively small sample sizes within some injury subgroups, particularly among female athletes. These results point toward a potentially complex and sex-specific relationship between AT stiffness and lower body injury, but do not allow for definitive conclusions. On the contrary, no differences were found between the previously injured group and the non-injured group in AT thickness or EI.

Numerous studies have examined the direct relationship between US properties and AT injuries, focusing primarily on tendinopathy and tendon rupture injuries. These have consistently reported a significant reduction in stiffness and EI, as well as an increase in thickness associated with both conditions (36, 69–71). Our results extend this evidence by suggesting that broader lower-limb injury Hx, not limited to tendon-specific pathology, may also be associated with alterations in AT stiffness. However, the inclusion of heterogeneous injury types and limited subgroup sizes may partially explain the absence of associations between AT thickness or EI and injury Hx.

These observations warrant further investigation using larger sample sizes within sex- and injury-specific subgroups to obtain more comprehensive assessments of the relationship between injury and AT properties. Future studies could, for example, track SWE, thickness, and EI measurements longitudinally across an athletic season, throughout recovery phases, and following bouts of exercise.

#### 4.1.4 Relation between fibrillar pattern and US properties and functional outcomes

Tendons with disrupted fibrillar architecture, which we defined as fragmentation, interruption, or loss of the normal linear fibrillar pattern, showed no differences in US-derived properties or in objective measures of functional performance in comparison to ATs with normal, organized fibrillar patterns. While most research has focused on the fibrillar pattern after AT reconstructive surgery, to our knowledge, no prior studies have evaluated this property as a determinant of structural characteristics and functional outcomes in athletic populations.

Emerging imaging evidence offers relevant insights. A recent study investigating tendon structure in healthy and symptomatic cohorts demonstrated that six weeks of ballet training induced an increase in fibrillar disorganisation without associated changes in clinical symptoms or functional performance (51). In contrast, longitudinal imaging in elite football players showed that the fibrillar pattern of asymptomatic AT's improved across a 5-month pre-season, with increases in aligned fibrils and decreases in disorganized echo-types (72). Together, these findings suggest that AT fibrillar pattern is dynamic and responsive to load but may not directly determine functional outcomes or other US-derived properties.

#### 4.1.5 Correlation between AT stiffness, thickness, and EI

Within the US-derived measures assessed, only thickness and EI demonstrated a consistent association. In both males and females, this relationship was characterized by a strong negative correlation, suggesting that increased thickness may be associated with reduced EI. A potential explanation is that greater thickness reflects pathological changes, such as fiber disorganization and elevated interstitial fluid, which contribute to decreased EI.

The current evidence supporting this association remains inconclusive. For instance, in asymptomatic pre-professional dancers, AT thickness did not differ between groups with distinct EI patterns (25). In contrast, a study in badminton players, which included both tendinopathic and healthy tendons, reported greater stiffness in ATs with more heterogeneous echogenicity, which the authors suggested could represent a transition from a physiological to a pathological state (73).

Overall, these findings indicate that the relationship between thickness and EI is context-dependent, with stronger associations observed in the presence of disease or injury.

Although mean EI provides a well-established and reproducible measure of tendon echogenicity, it represents a global summary of grayscale values and does not capture spatial heterogeneity within the tissue. Advanced texture-based US metrics, such as entropy, contrast, or correlation, may offer additional insight into tendon microstructural organization by quantifying grayscale variability and spatial patterns. The inclusion of such texture analyses was beyond the scope of the present study but represents an important avenue for future research aimed at further characterizing tendon structural adaptations in athletic populations.

#### 4.1.6 Correlation between ankle ROM and AT stiffness, thickness, and EI

Unlike prior work, our findings did not reveal a significant correlation between AT stiffness and ankle ROM (19). A recent study using SWE and a digital goniometer-based lunge test in 20 healthy young individuals reported a moderate negative correlation between ankle dorsiflexion ROM and AT stiffness (19). Several methodological differences may explain the discrepancy with our results: the participants involved in their study were non-athletes, their ROM testing procedures differed from our knee-to-wall test, and they only performed SWE on the right limb of the participants (19). These discrepancies make direct comparison less reliable.

Although the AT plays a role in ankle ROM based on its anatomical insertion, several other factors also influence ankle ROM, including joint capsule stiffness, sex, age, pain and stretch tolerance, Hx of ankle injury, and the athlete's warm-up state (18, 74–76). These influences likely dilute the role of AT stiffness, helping explain the absence of significant correlations in our cohort.

Support for this interpretation comes from related tendon and muscle studies. Gastrocnemius EI has shown no correlation with maximal dorsiflexion ROM (74), and AT thickness likewise shows no association with ROM in young collegiate athletes (77). Taken together, these findings suggest that structural properties of the tendon and muscle alone may not be sufficient to explain individual differences in ankle dorsiflexion. Instead, ankle ROM should be understood as an emergent property shaped by a broader set of biomechanical and neuromuscular factors.

#### 4.1.7 Correlation between performance measures and US properties

Previous studies have demonstrated that greater functional stiffness in the lower limb can enhance performance (3, 14, 15). However, in our cohort, local AT stiffness, thickness, and EI did not correlate with jump height. Although the type of jump used in our study differed from those in earlier reports, our findings are consistent with two previous studies that reported no correlation between AT stiffness and jump height (78, 79). These studies analyzed the tendons' behaviour during physical tasks to deduce a measure of functional tendon stiffness, rather than SWE, and employed slightly different jump test protocols, yet yielded similar results (78, 79).

The lack of association is likely explained by the multifactorial nature of jump height (80). Age, sex, body composition, power output, and jumping technique all influence jump performance (81–84). While tendon properties may contribute, their relative effect is likely outweighed by these other variables. In particular, EI appears to have little predictive value for functional outcomes in healthy or athletic populations (25). Therefore, these US-derived properties may not be major determinants of jump height, which is a complex task requiring contributions from multiple muscle groups and a high degree of coordination.

A novel aspect of our study was the exploration of correlations between AT properties and HR test outcomes (repetition count and total HR height). It was initially hypothesized that these outcome measures would reflect the strength of the ankle plantar flexor muscle-tendon unit and correlate with AT US-derived health indicators (85). However, our findings did not support this assumption. One explanation is that the HR test may be a more accurate indicator of muscular endurance than strength. Endurance performance is shaped by a range of physiological and neuromuscular factors, including body composition, training Hx, oxygen uptake, blood flow, capillary density, mitochondrial capacity, and preferential recruitment of type I fibers (86–90). All of which were not assessed and may have influenced the results.

### 4.2 Limitations

Although our sample size was comparable to that of previous SWE AT-based investigations involving university and professional-level athletes (10, 91), the relatively small size still presents a limitation to this study, particularly among female athletes, which may have limited statistical power and necessitates cautious interpretation of injury-related and sex-specific findings. Twenty males and 11 female players initially volunteered for both the SWE imaging and physical performance measurements, but only 9 female participants completed the performance assessments (jump and HR tests) due to constraints imposed by the team's coaches. These constraints were primarily related to injury prevention and reluctance to have players fatigued due to the testing date being scheduled too close to the start of the competitive season. A larger sample size may have revealed a statistically significant difference between our male and female players' AT stiffness levels.

Another limitation relates to the inherent variability of US imaging-derived outcomes when certain confounding factors are not adequately controlled. Although participants were instructed to refrain from engaging in physical activity prior to testing, their general daily activity levels pre-testing were not monitored. Routine movements throughout the day may influence AT stiffness, as increased tendon temperature resulting from activity can lead to elevated SWE values (41). To mitigate this, all measurements were conducted in the morning to ensure consistency. Despite instructing participants to remain as still as possible during SWE imaging, minor muscle spasms, respiration, and general restlessness can also contribute to possible measurement variations (27).

Furthermore, ankle dorsiflexion ROM was assessed using a distance-based, weight-bearing knee-to-wall test. Although this functional approach is widely used and clinically relevant, the linear distance measured may be influenced by individual foot and lower-limb anthropometric characteristics, such as foot length or segment proportions. This consideration may be particularly relevant in a basketball population, which is characterized by greater variability in stature and foot size. As these variables were not directly assessed or normalized, their potential influence on ROM measurements cannot be fully excluded. However, given the bilateral testing approach and the relatively homogeneous training background of the cohort, anthropometric effects are unlikely to fully explain the absence of significant associations between ankle ROM and AT mechanical properties.

Finally, the timing of data collection represents an additional limitation. Due to technical issues with the US machine during the pre-season period, access to the device was delayed until the latter part of the pre-season. Consequently, testing for the women's basketball team was conducted during this window, while testing for the men's team was postponed until mid-season break. These time-based discrepancies in testing may have influenced AT stiffness, thickness, and EI values, particularly for the male participants, who may have experienced greater cumulative tendon loading by mid-season.

### 4.3 Implications for future research

Future research should prioritize larger sample sizes and longitudinal study designs to better characterize how AT stiffness, thickness, and EI evolve over time and in response to training, performance demands, and injury exposure. This would be particularly valuable for clarifying the sex-specific and injury-related trends suggested by the present findings, which were limited by small subgroup sizes.

Continued efforts to standardize and report methodological parameters, such as ankle positioning, probe orientation, ROI definition, and pre-assessment activity levels, remain essential to improve reproducibility and comparability across SWE-based studies. In addition, the consistent association observed between AT thickness and EI in the present study highlights the need for further investigation into the structural mechanisms linking these variables across different athletic and clinical contexts.

Finally, the absence of associations between AT mechanical properties and functional performance outcomes suggests that future studies may benefit from incorporating more direct measures of plantar flexor strength. This could help disentangle the relative contributions of tendon structure, muscle capacity, and movement strategy to performance and injury risk.

## 5 Conclusion

This study examined the relationship between AT US-derived properties, leg dominance, and functional measures in university-level basketball players. Although no statistically significant associations were found between AT US properties, performance, or ROM measures, several trends emerged. Notably, injury Hx significantly affected tendon stiffness, and strong positive correlation between AT thickness and EI. This study contributes to the growing body of research on tendon properties in athletic populations and emphasizes the importance of considering sex, injury status, and methodological consistency when interpreting US-derived values. Future longitudinal research involving larger cohorts and continuous monitoring across an entire athletic season would be essential. Such studies would enhance the understanding of how tendon properties evolve. They may contribute to the development of preventive, treatment, and training techniques to reduce the risk of injuries and improve performance.

## Data Availability

The original contributions presented in the study are included in the article, further inquiries can be directed to the corresponding author.
